# Trend change analysis of postural balance in Parkinson’s disease discriminates between medication state

**DOI:** 10.1186/s12984-024-01411-z

**Published:** 2024-06-28

**Authors:** Piotr Wodarski, Jacek Jurkojć, Marta Chmura, Elke Warmerdam, Robbin Romijnders, Markus A. Hobert, Walter Maetzler, Krzysztof Cygoń, Clint Hansen

**Affiliations:** 1 Faculty of Biomedical Engineering, Department of Biomechatronics, Silesian University of Technology, Gliwice, Poland; 2 Division of Surgery, Saarland University, 66421 Homburg, Germany; 3 Department of Neurology, Kiel University, 24105 Kiel, Germany; 4 Skyfi Sp. z o.o, Gliwice, Poland

**Keywords:** Parkinson Disease, Trend Change Index, Body balance, Postural Stability, Balance, Wearable sensors, Neurology

## Abstract

**Background:**

Maintaining static balance is relevant and common in everyday life and it depends on a correct intersegmental coordination. A change or reduction in postural capacity has been linked to increased risk of falls. People with Parkinson’s disease (pwPD) experience motor symptoms affecting the maintenance of a stable posture. The aim of the study is to understand the intersegmental changes in postural sway and to apply a trend change analysis to uncover different movement strategies between pwPD and healthy adults.

**Methods:**

In total, 61 healthy participants, 40 young (YO), 21 old participants (OP), and 29 pwPD (13 during medication off, PDoff; 23 during medication on, PDon) were included. Participants stood quietly for 10 s as part of the Short Physical Performance Battery. Inertial measurement units (IMU) at the head, sternum, and lumbar region were used to extract postural parameters and a trend change analysis (TCA) was performed to compare between groups.

**Objective:**

This study aims to explore the potential application of TCA for the assessment of postural stability using IMUs, and secondly, to employ this analysis within the context of neurological diseases, specifically Parkinson’s disease.

**Results:**

Comparison of sensors locations revealed significant differences between head, sternum and pelvis for almost all parameters and cohorts. When comparing PDon and PDoff, the TCA revealed differences that were not seen by any other parameter.

**Conclusions:**

While all parameters could differentiate between sensor locations, no group differences could be uncovered except for the TCA that allowed to distinguish between the PD on/off. The potential of the TCA to assess disease progression, response to treatment or even the prodromal PD phase should be explored in future studies.

**Trial registration:**

The research procedure was approved by the ethical committee of the Medical Faculty of Kiel University (D438/18). The study is registered in the German Clinical Trials Register (DRKS00022998).

**Supplementary Information:**

The online version contains supplementary material available at 10.1186/s12984-024-01411-z.

## Introduction

Maintaining an upright posture, or static balance, is a fundamental aspect of human life that underscores the intricate interconnections of the vestibular, visual, and somatosensory systems within the central nervous system [1]. Posture is more than the mere static alignment of body segments; it represents a dynamic process characterized by continuous adjustments to maintain stability while performing various tasks. Maintaining upright posture becomes increasingly critical with aging and neurological disorders due to the gradual decline in postural control, predisposing individuals to an elevated risk of falls and associated injuries. This decline is influenced by a multitude of factors, encompassing alterations in sensory input, muscle strength, joint flexibility, and neural processing [2]. As an example pwPD present profound challenges to postural control [3] which is based on the neurodegenerative character of the disease characterized by the loss of dopaminergic neurons. The difficulties with balance are linked to the loss of dopaminergic neurons affecting the basal ganglia which are essential to control upright posture.

A particularly intriguing aspect of postural control is the necessity for specific body segments to remain stable while others adapt to accommodate external demands. For instance, the head must remain stable to preserve visual focus and spatial orientation [4], while the pelvis may need to make adjustments to accommodate changes in terrain or task requirements [5]. Unconsciously, humans stabilize their visual focus or gaze and maintain awareness of their body position [6] but also stabilize their head to ensure balance [7]. For example Wallard et al. [8] found that children with cerebral palsy exhibit greater head angle variability, suggesting a compensatory strategy and Pozzo et al. [5] observed significant head stabilization during various locomotor tasks, with the head compensating for translation and rotation. People with mild traumatic brain injury revealed increased sway of the center of mass and less head stabilization compared with healthy controls [9]. In addition Israeli-Korn et al. [10] showed that intersegmental coordination patterns differ e.g. between Parkinson’s disease and cerebellar ataxia. Honegger et al. [11] investigated the coordination of the head with respect to the trunk, pelvis, and lower leg during quiet stance after vestibular loss. They argue that such simplification, as proposed by Fitzpatrick et al. [12] and Pinter et al. [13], may not fully capture the complexity of postural control in these populations. Contrary to expectations, their findings reveal synchronous movements of the head and trunk among healthy controls, suggesting that the presence of an intact vestibular system does not necessarily confer greater stability to the head in space. Instead, the pelvis emerges as a key stabilizing factor, as supported by earlier studies [13, 14] and the present investigation. These studies collectively highlight the role of aligning of body segments in postural control, particularly in individuals with motor impairments introducing another layer of complexity to our understanding of static balance. This raises the question of how the body segments sway and are controlled within the realm of quiet stance in different pathologies.

Inertial measurement units (IMUs) are small body-mounted sensors containing accelerometers, gyroscopes and magnetometers that can track 3D human movement on a very granular level e.g. to measure balance [15, 16] based on center of mass movements [17, 18]. Their reliability and validity have been extensively examined [19, 20] and provide a tool to be used in combination with a trend change analysis (TCA) [21]. TCA can detect the small number of quick corrections, an increased frequency of longer-duration corrections, and an elongation in the displacement between successive postural corrections. Adapted from techniques originally employed in stock exchange analyses, the TCA facilitates the quantification of postural corrections in both the anteroposterior (A/P) and mediolateral (M/L) directions. Moreover, it allows for the calculation of the number of adaptations, the time interval between successive posture corrections [21] providing insights about the body’s responses to postural challenges [22].

The research presented herein aims to delve into the intricate relationship between maintaining an upright posture, PD, aging, and the dynamic adjustments involving intersegmental control. The objectives of this study are twofold: Firstly, to explore the potential application of TCA for the assessment of postural stability using IMUs, and secondly, to employ this analysis within the context of neurological diseases, specifically PD. We hypothesized that the TCA could differentiate between persons with PD (pwPD) and healthy adults and also distinguish, in pwPD, between dopaminergic on (PDon) and dopaminergic off phases (PDoff).

## Methods

### Participants

The experimental groups consisted of 61 healthy participants, 40 young (YO), 21 old (OP) and 29 pwPD. The demographic characteristics of the study participants are presented in Table 1.

**Table 1:** Characteristics of study participants (YO: young, OP: old, pwPD: persons with PD, w: women, m: men)

	YO	OP	pwPD
*N (w/m)*	40 (20/20)	21 (11/10)	29 (18/11)
*Age(w/m) [year]*	29.5 ± 8.5 / 27.5 ± 7.1	72.5 ± 5.9 / 70.9 ± 6.0	63.2 ± 11.7 / 68.0 ± 7.3
*Weight (w/m) [kg]*	79.5 ± 11.5 / 66.3 ± 8.5	83.9 ± 13.3 / 68.9 ± 12.5	88.5 ± 15.3 / 69.3 ± 14.4
*Height (w/m) [m]*	1.85 ± 0.08 / 1.73 ± 0.05	1.81 ± 0.08 / 1.66 ± 0.06	1.78 ± 0.07 / 1.67 ± 0.06

All participants were either inpatients at the neurogeriatric ward of the Neurology Center at the University Hospital Schleswig-Holstein, Campus Kiel, or spouses of the patients or members of the professional team. pwPD were diagnosed according to the Movement Disorder Society clinical diagnostic criteria for Parkinson’s disease [23, 24]. Thirteen pwPD participated as PDoff (UPDRS III score 24 ± 10), 23 as PDon (UPDRS III score 30 ± 20), and 7 as both PDon (UPDRS III score 26 ± 10) and PDoff (UPDRS III score 27 ± 10). The sample size for this study was predetermined based on prior research and the current analysis is a secondary analysis of the previously published data set [25–27].

The study was conducted according to the guidelines of the Declaration of Helsinki and approved by the Ethics Committee of Kiel University (D438/18) and all participants provided written informed consent before participation. Participants were excluded when their fall risk was determined to be too high (> 2 falls in the previous week), corrected visual acuity was below 60%, they scored ≤ 15 points in the Montreal Cognitive Assessment (MoCA) test [24, 28], had current or past chronic substance abuse (except nicotine), and were not able to perform at least one of the walking tasks [25].

### Protocol

Data from the IMU sensors were recorded using a motion capture system (Noraxon USA Inc., myoMOTION 3.16, Scottsdale, AZ, USA) [25, 26]. The participants were asked to stand in an upright position with their feet together, side-by-side and fix their gaze on a point on a white wall for 10 s as part of the Short Physical Performance Battery [25].

**Fig. 1:**
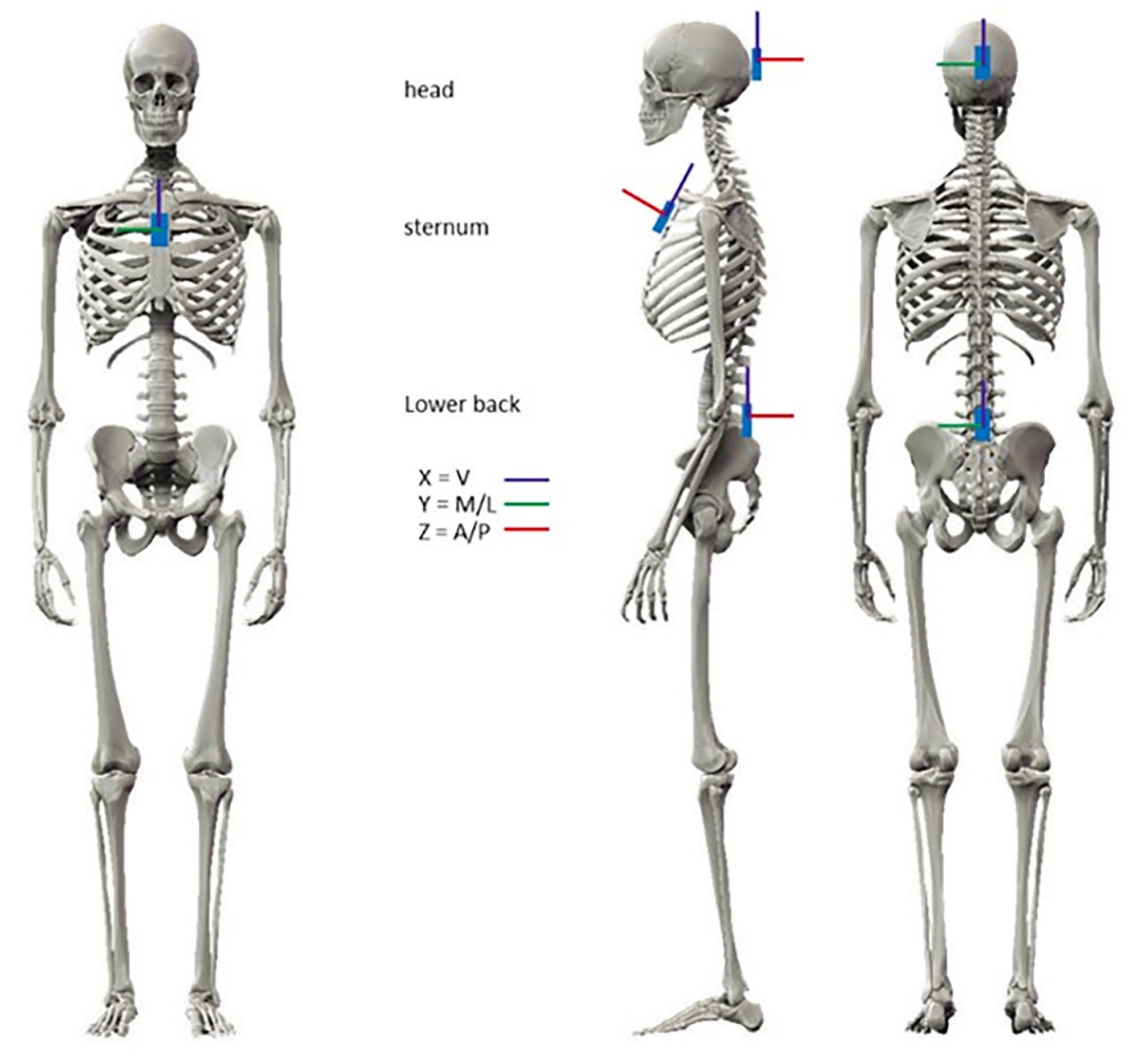
Placement of the inertial measurement units on the head, sternum and pelvis

Three IMUs were attached to the body (pelvic, sternum and head) using elastic bands with a special housing for the IMU to clip into (see Fig. 1). The research procedure was approved by the ethical committee of the Medical Faculty of Kiel University (D438/18). The study is registered in the German Clinical Trials Register (DRKS00022998).

### Sensor data processing

The IMU data was processed by custom written scripts using MATLAB (MathWorks, Nantick, MA) based on methodology described by Mancini et al. [29]. The parameters provided information about the sway jerkiness (JERK) (cm^2^/s^5^), the sway area (SURFACE) (cm^2^), path (PATH) (cm), mean velocity (MV) (cm/s), range of acceleration (RANGE) (cm/s^2^) and root mean square of the acceleration (RMS) (cm/s^2^).

In addition, the TCA was applied. Acceleration signals were filtered with a low-pass filter (7 Hz low-pass Butterworth filter). The method is based on a Moving Average Convergence Divergence (MACD) indicator calculation algorithm and evaluates the relationships of exponential moving averages (EMAs) for the recorded signal [21]. Calculations can be performed for any time-varying signal. In the case of the tests used, recorded acceleration signals were used, the S signal is the acceleration signal.

In the first step of calculations, for the signal S, the MACD line was determined as the difference between two EMAs (Eq. 2) with lengths of 12 and 26 samples according to Eq. 1.

Eq. 1 $$MACD=EM{A}_{S,12}- EM{A}_{S, 26}$$

Where EMA_S_,_12_ - faster exponential moving average for signal S,

EMA_S,26_ - slower exponential moving average for signal S

Eq. 2 $$EMA=\frac{{p}_{0}+ \left(1-\alpha \right){p}_{1}+{\left(1-\alpha \right)}^{2}{p}_{2}+\dots + {\left(1-\alpha \right)}^{N}{p}_{N}}{1+\left(1-\alpha \right)+{\left(1-\alpha \right)}^{2}+\dots +{\left(1-\alpha \right)}^{N}}$$

Where, p_0_ – ultimate value, p_1_ – penultimate value, p_N_ – value preceding N periods, N = number of periods, α = a smoothing coefficient equal to 2/(*N* + 1).

In the next step, the signal line is calculated as an EMA with a length of 9 samples from the MACD line signal in accordance with Eq. 3.

Eq. 3 $$Signal\, line =EM{A}_{MACD line, 9}$$

The intersection of the MACD line and the Signal line determines the trend change points in the S signal. The number of intersections determines the TCI (trend changes index).

**Fig. 2:**
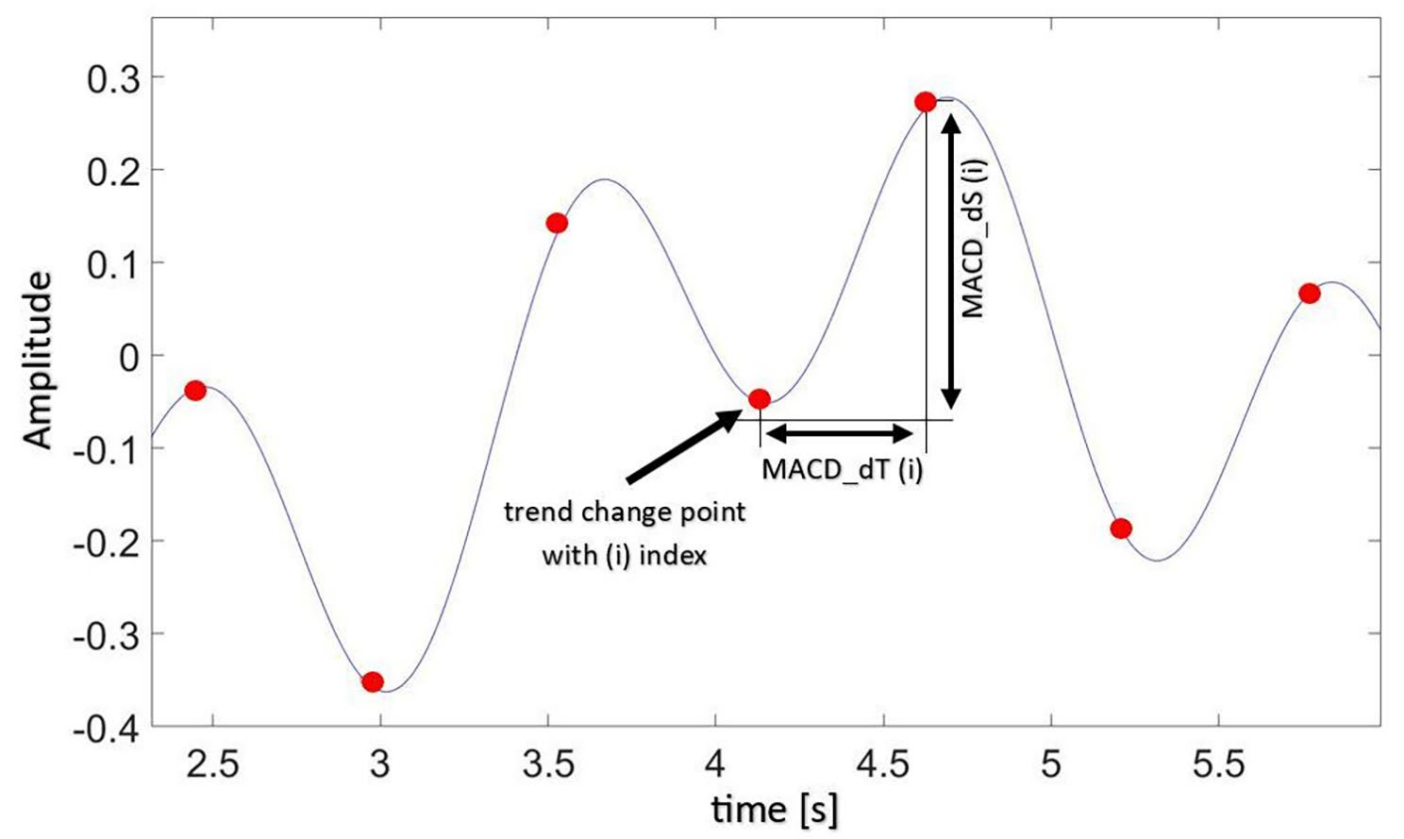
Graphical explanation of the Trend Change Index (TCI), the delta time between successive TCIs (MACD_dT) as well as the delta space between successive TCIs (MACD_dS) in an acceleration signal from a sensor on the pelvis with an observation phase of about 3 s. Seven trend changes (indicated by the seven red dots) are shown. All determined MACD_dTs were used to calculate TCI_dT and all MACD_dSs to calculate TCI_dS according to the procedure described in the text

In the next step, the time intervals between successive points of trend changes in the S signal were calculated. In this way, the MACD_dT array was determined, the average value of which is the value of the TCI_dT. As a consequence, the displacement between subsequent trend change points were calculated and the results constitute the MACD_dS array. The average value of the array is the value of the TCI_dS (Fig. 2). Finally, the corresponding elements of the MACD_dS array were divided by MACD_dT to obtain the MACD_dV array. The average value of the array is the value of the TCI_dV. In this study, the displacement of the signal is the difference in the acceleration values between successive points of trend change on the acceleration signal.

To summarize, TCI determines the number of trend changes in the assumed research period, TCI_dT defines the average time between detected trend changes, and TCI_dS determines the average value of the acceleration change between subsequent trend changes. Indices were determined for each of the three directions of measurement, and then the resultant values were determined i.e. for TCI as the sum of the number of trend changes detected in each direction of the measured accelerations (in the X, Y and Z axes), and for TCI_dT, TCI_dS, TCI_dV as the square root of the sum of squares of the values calculated in each direction.

### Statistical analysis

The analyses were performed using Matlab R2022a and JASP (Version 0.16.1 JASP Team (2022)) for all statistical analyses.

The analysis aimed to investigate differences between sensor positions and cohorts within the dataset. Shapiro-Wilk tests revealed significant deviations from normality (*p* < 0.05) across multiple groups and sensor positions, thus prompting the utilization of non-parametric tests. Subsequently, a Kruskal-Wallis H Test were employed to evaluate variations between cohorts and sensor positions. In case of statistically significant differences (*p* < 0.05) post-hoc analyses, utilizing Dunn’s test with Bonferroni correction, were conducted to ascertain specific group disparities.

## Results

When comparing the individual parameters for each sensor and each cohort (Table 2), no differences could be found between the cohorts but significant differences were uncovered between the sensor positions (Additional file 1).

**Table 2:** Values of individual parameters (M - median, QR/2 - the half of coefficient quartile of variation)

		Head				Pelvis				Sternum			
		YO	OP	PD on	PD off	YO	OP	PD on	PD off	YO	OP	PD on	PD off
JERK [cm2/s5]	**M**	30.78	37.54	28.52	28.06	7.64	10.48	8.28	8.08	9.22	12.12	10.26	8.59
	**QR/2 [%]**	190	123	56	60	58	54	67	59	109	58	55	50
MV [cm/s]	**M**	24.17	29.66	26.50	26.97	12.15	13.32	14.39	15.57	14.25	16.78	16.80	15.98
	**QR/2 [%]**	38	29	22	24	31	34	29	31	31	21	17	24
PATH [cm]	**M**	241.69	296.59	265.04	269.73	121.47	133.16	143.92	155.67	142.52	167.84	168.02	159.75
	**QR/2 [%]**	38	29	22	24	31	34	29	31	31	21	17	24
RMS [cm/s2]	**M**	2.72	3.18	2.11	3.13	0.91	1.21	1.14	1.17	1.15	1.47	1.43	1.34
	**QR/2 [%]**	67	72	83	36	25	34	38	14	44	41	38	29
SURFACE [cm2/s4]	**M**	35.96	81.86	35.94	71.10	7.09	10.40	10.90	11.22	10.49	18.07	17.19	14.17
	**QR/2 [%]**	229	145	209	74	49	77	48	29	89	84	70	61
RANGE [cm/s2]	**M**	10.73	12.02	7.83	9.38	2.97	4.29	4.19	3.38	3.43	5.61	4.37	4.46
	**QR/2 [%]**	78	89	64	73	41	52	44	15	72	53	43	24
TCI [no]	**M**	258	272	269	302	295	295	284	317	296	301	287	310
	**QR/2 [%]**	9	5	4	4	4	6	7	5	3	5	7	2
TCI_dT [s]	**M**	0.21	0.20	0.19	0.17	0.18	0.18	0.18	0.16	0.18	0.17	0.18	0.17
	**QR/2 [%]**	8	4	5	4	4	6	9	5	4	5	6	2
TCI_dS [cm]	**M**	2.08	2.29	1.89	1.76	0.88	0.74	1.02	0.97	1.01	0.96	1.02	1.02
	**QR/2 [%]**	59	40	35	23	36	46	44	27	35	36	35	18
TCI_dV [cm/s]	**M**	15.63	19.49	15.90	17.22	8.26	6.90	9.59	9.20	9.32	8.45	11.18	10.04
	**QR/2 [%]**	48	32	30	25	34	56	39	26	35	44	25	19

The sensor position differed for all cohorts and all parameters except TCI and TCI_dT for PDon (Table 3).

**Table 3:** Sensor parameters to differentiate between groups and sensor positions in controls and PDon. The H-statistics of the Kruskall-Wallis test as well as the degree of freedom and significance levels are reported within the tables

Parameters	Group level	Sensor position	YO post hoc *p* < 0.05	OP post hoc *p* < 0.05	PDon post hoc *p* < 0.05
JERK	n.s.	H(2) = 60.29, *p* < 0.001	head vs. sternum and pelvis	head vs. sternum and pelvis	head vs. sternum and pelvis
MV	n.s.	H(2) = 70.87, *p* < 0.001	head vs. sternum and pelvis	head vs. sternum and pelvis	head vs. sternum and pelvis
PATH	n.s.	H(2) = 70.87, *p* < 0.001	head vs. sternum and pelvis	head vs. sternum and pelvis	head vs. sternum and pelvis
RMS	n.s.	H(2) = 73.18, *p* < 0.001	head vs. sternum and pelvis	head vs. sternum and pelvis	head vs. sternum and pelvis
SURFACE	n.s.	H(2) = 69.59, *p* < 0.001	head vs. sternum and pelvis	head vs. pelvis	head vs. sternum and pelvis
RANGE	n.s.	H(2) = 54.82, *p* < 0.001	head vs. sternum and pelvis	head vs. pelvis	head vs. sternum and pelvis
TCI	n.s.	H(2) = 44.27, *p* < 0.001	head vs. sternum and pelvis	head vs. sternum and pelvis	
TCI_dT	n.s.	H(2) = 57.37, *p* < 0.001	head vs. sternum and pelvis	head vs. sternum and pelvis	
TCI_dS	n.s.	H(2) = 79,63, *p* < 0.001	head vs. sternum and pelvis	head vs. sternum and pelvis	head vs. sternum and pelvis
TCI_dV	n.s.	H(2) = 58.94, *p* < 0.001	head vs. sternum and pelvis	head vs. sternum and pelvis	head vs. sternum and pelvis

When comparing the PDon and PDoff cohort (Table 4) only TCI & TCI_dT differed between the PDon and PDoff cohort. Significant differences were found between the three sensor locations (Table 5).

**Table 4:** Values of parameter for 7 pwPD tested “on” and “off” (M - median, QR/2 - the half of coefficient quartile of variation)

		Head		Pelvis		Sternum	
		PD off	PD on	PD off	PD on	PD off	PD on
JERK [cm2/s5]	**M**	34.71	44.54	13.08	8.07	8.87	10.26
	**QR/2 [%]**	67	27	79	6	267	39
MV [cm/s]	**M**	32.82	29.79	18.66	14.39	16.57	16.80
	**QR/2 [%]**	17	15	28	6	59	10
PATH [cm]	**M**	328.20	297.90	186.62	143.92	165.71	168.02
	**QR/2 [%]**	17	15	28	6	59	10
RMS [cm/s2]	**M**	3.27	3.67	1.23	1.53	1.84	1.69
	**QR/2 [%]**	21	51	11	21	28	16
SURFACE [cm2/s4]	**M**	77.93	113.99	11.33	12.86	27.54	24.67
	**QR/2 [%]**	55	81	31	21	44	35
RANGE [cm/s2]	**M**	13.87	10.17	3.35	4.27	5.20	5.75
	**QR/2 [%]**	40	83	15	33	21	14
TCI [no]	**M**	308	275	316	279	310	277
	**QR/2 [%]**	3	3	4	6	2	3
TCI_dT [s]	**M**	0.17	0.19	0.16	0.19	0.17	0.19
	**QR/2 [%]**	4	3	5	5	2	3
TCI_dS [cm]	**M**	1.85	2.71	1.11	1.05	1.10	1.27
	**QR/2 [%]**	21	26	26	18	26	23
TCI_dV [cm/s]	**M**	20.48	17.68	11.55	9.77	10.68	11.18
	**QR/2 [%]**	15	27	32	8	27	19

**Table 5:** Sensor parameters to differentiate between groups and sensor positions in PDon and PDoff. The H-statistics of the Kruskall-Wallis test as well as the degree of freedom and significance levels are reported within the tables

Parameters	Group level	Sensor position
JERK	n.s.	H(2) = 12.63, *p* = 0.002
MV	n.s.	H(2) = 11.11, *p* = 0.004
PATH	n.s.	H(2) = 11.11, *p* = 0.004
RMS	n.s.	H(2) = 13.09, *p* = 0.001
SURFACE	n.s.	H(2) = 17.12, *p* < 0.001
RANGE	n.s.	H(2) = 11.59, *p* = 0.003
TCI	H(2) = 13.40, *p* < 0.001	n.s.
TCI_dT	H(2) = 13.21, *p* < 0.001	n.s.
TCI_dS	n.s.	H(2) = 9.13, *p* = 0.01
TCI_dV	n.s.	H(2) = 7.59, *p* = 0.022

## Discussion

This study investigated postural stability of healthy young, old controls and persons with PD in a static balance task using three different sensor locations. The aim of the study was to analyze the upright posture and intersegmental adjustments, to evaluate whether the parameters could uncover distinct postural sway behavior between the different cohorts. Our results confirmed that both, the postural parameters and TCA, could uncover sway differences between the segments but only the TCA could differentiate between PDon and PDoff.

The results of the current study show no group differences between the healthy adults and pwPD, confirming results from a previous study investigating static sway with increasing task difficulty [27]. This is of interest as PD is known for its altered postural reflexes with a disruption of the precisely coordinated execution of agonist and antagonist muscles (associated with bradykinesia and rigidity), which results in difficulty to maintain static postural stability [30–32] due to a reduced margin of stability [33].

While pwPD have shown larger values for sway acceleration, jerk and sway velocity during postural balance compared to age-matched healthy controls [29, 34] they also show an increased jerkiness during the performance of cognitive task [35], suggesting an interaction of cognitive functions, including multisensory integration, with static balance mechanisms. Our results highlight larger motions from the head compared to the sternum and the pelvis. The results convey with previous findings [14] basing their findings upon the biomechanical principal of a double-inverted pendulum. The double-inverted pendulum allows to be controlled by the ankles, the hip or both, while assuming a rigid head-on-trunk coupling. Almost all parameters were able to distinguish between sensor position indicating the complex relationship between the dynamic intersegmental adjustments and upright posture. The results suggest that for a relative simple and short balance tasks pwPD can perform control-like, which could be related to the location of the pathology within the central nervous system and its extensive compensation possibilities [36] and by using alternative pathways or even networks [37].

There is some evidence that dopaminergic medication can improve static sway [38, 39]. However, there are not many IMU-based studies available that can show these differences. One reason may be that the parameters currently assessed for this performance are not covering disease-relevant changes. Here we introduced TCA in the analysis of static sway in PDon and PDoff, and could in fact detect significant differences only with this approach (but not with the conventional parameters). We found a higher number of TCIs and smaller TCI_dT values in PDoff compared to PDon. This is coherent with previous results obtained for COP measurements showing an increase in TCIs and reduction of TCI_dT in pwPD compared to healthy individuals [40]. In our view, this perspective also aligns with a pathomechanistic standpoint. Previous research, as indicated by Bizid et al. [41], suggests that low frequencies are predominantly associated with visuo-vestibular regulation, while high frequencies are associated with proprioceptive regulation. Additionally, it is well-established that visual perception and integration are strongly dopamine-dependent [42]. Therefore, we hypothesize that the results observed through TCA most likely reflect visual deficits resulting from a dopaminergic deficit. This is particularly evident, given that lower leg proprioceptive performance does not appear to be influenced by dopaminergic treatment [43].

### Limitations

It would be worthwhile to mention limitations of the current study. First, the number of pwPD measured in both medication states was relatively low, potentially limiting the generalizability of findings and the ability to capture the full spectrum of balance-related issues in PD. Another constraint lies in the brief 10-second measurement duration, which may not provide a comprehensive representation of individuals’ balance control capabilities, particularly in dynamic real-world scenarios. Additionally, the use of a side-by-side stance as a measure may cause limitations as it may not be challenging enough to detect subtle differences between cohorts or uncover changes in postural control based on intersegmental coordination. These limitations emphasize the need for cautious interpretation of results and highlight areas for future research to address these constraints and provide a more nuanced understanding of balance control in Parkinson’s disease and other relevant populations. Nevertheless, considering these limitations, it is all the more remarkable given that the TCA parameters were effective in distinguishing between PD on and PD off.

### Clinical implication

This study investigated static sway in healthy individuals and pwPD using three sensor locations. Results show that postural parameters effectively distinguish between segments. However, and even more relevant, the introduction of TCA proves instrumental in detecting significant differences between PDon and off medication, showcasing its potential in assessing disease-relevant changes not captured by conventional parameters.

### Electronic supplementary material

Below is the link to the electronic supplementary material.

Supplementary Material 1

## Data Availability

No datasets were generated or analysed during the current study.
